# Development and application of a binary medium bond contact model for calcareous sands

**DOI:** 10.1371/journal.pone.0319014

**Published:** 2025-02-28

**Authors:** Qifen Cheng, Ge Zhang

**Affiliations:** 1 Key Laboratory of Geological Hazards on Three Gorges Reservoir Area, Ministry of Education, China Three Gorges University, Yichang, Hubei, China; 2 College of Civil Engineering & Architecture, China Three Gorges University, Yichang, China; Ural Federal University named after the first President of Russia B N Yeltsin Institute of Physics and Technology: Ural'skij federal'nyj universitet imeni pervogo Prezidenta Rossii B N El'cina Fiziko-tehnologiceskij institut, RUSSIAN FEDERATION

## Abstract

In the construction process of islands and reefs in the South China Sea, calcareous sand, as an important foundation and building material, has been widely used in engineering practice. It is crucial to explore the mechanical properties of calcareous sands to ensure the stability and maintenance of these islands and reefs. To study the effects of the bonding progressive failure on its macroscopic mechanical properties, a binary media bonding contact model is developed. This model can describe the gradual deterioration process of the intergranular cementation. Additionally, a discrete elemental contact subroutine (DLL) is created by C++ language program for the particle flow program (PFC2D) to utilize. The compression and direct shear testing of single contact was subjected. The computational accuracy of the proposed binary media contact model was confirmed by the comparison of the theoretical and numerical results. The biaxial shear discrete element simulations were conducted to study the behaviors of calcareous sand under different confining pressure conditions. The stress-strain curves obtained are in good agreement with the experimental results. The results show that the proposed binary media bonding contact model can accurately reflects the mechanical properties of calcareous sand. Based on calibrated discrete element contact mesoscopic parameters, the biaxial shear discrete element numerical simulations with different damage parameters were carried out. The effects of damage parameters on the stress-strain curves and shear strength are discussed. The evolution of effective coordination number, fabric anisotropy and force chain number are also analyzed. The research results can provide a basis for the study of macro-micromechanical properties of calcareous sand

## 1. Introduction

There is still a huge space for the development and utilization of marine resources. Many countries have begun to promote the development and utilization of marine resources in an all-round and in-depth way. There are a number of marine engineering projects, such as offshore oil extraction platforms, cross-sea bridges, sea airports, and underwater tunnels, that need to be built. Calcareous sand has been extensively utilized as a crucial foundation material in the construction process of China’s South China Sea islands and reefs project [1]. Calcareous sand is a marine geotechnical material that contains a significant amount of calcium carbonate, a type of insoluble carbonate [2–4]. The mechanical properties of Calcareous sand differ from typical land-based sand because of its biological formation [5–9]. As a result, The means of building construction on calcareous sand foundation is difficult to obtain from the existing construction experience, which restricts the further development of the island and reef project in the South China Sea. It is necessary to investigate the macroscopic failure mechanism of calcareous sand. Calcareous sand is characterized by irregular particle shape and high porosity. The cementation between calcareous sand particles is easily broken [10–13]. The failure criteria for particles bond is closely related to its macroscopic mechanical properties [14,15]. It is of great significance for the construction of island infrastructure to reveal the failure laws of intergranular meso-cementation.

At present, there are many researches have been carried out on the compression characteristics, shear characteristics and particle breakage characteristics of calcareous sand. By triaxial isotropic compression test, it indicated that the compression behaviors of calcareous sand specimens are greatly influenced by the initial pore ratio at low-stress levels. If the stress level reaches the yield stress, the compression properties are primarily governed by the particle crushing occurring within the specimen [16–18]. In the compression process, the overall rebound deformation is small due to the action of particle breakage. The non-recoverable plastic deformation accounts for the main part [16,19,20]. The test results show that calcareous sand exhibits low shear shrinkage during the initial stage under low pressure. Then, the significant shear expansion is occur during the later stage. Under the high confining pressure, the whole sample is easy to show shear shrinkage (strain hardening). Many interparticle cementation are broken, resulting in significant plastic deformation characteristics [21–24]. Although particle fragmentation has an impact on the mechanical behaviors of calcareous sand during shear process. It found that the particle fragmentation of calcareous sand specimens will not develop indefinitely by indoor tests. The degree of particle fragmentation will eventually converge to a stable value, which means that the mechanical behaviors of calcareous sand will no longer be affected by particle fragmentation [5,25–28].

To find out the influence of particle breakage characteristics on its mechanical behavior, Yu [29] presented the evolution law of particle breakage in the whole process of triaxial shear of calcareous sand. The adopted fractal model and various particle breakage indicators were put forward to give the quantitative evolution process of calcareous sand particle breakage. Peng [30] et al. used staining calibration and image particle segmentation methods to achieve accurate analysis of the absolute broken amount of calcareous sand with different particle sizes. Then, a new test method for the study of broken particle materials were proposed. To quantitatively describe the rule of particle breakage by mathematical methods, Zhang et al. [31] proposed a particle breakage evolution model based on Weibull distribution. Based on the stochastic process theory, Tong et al. [32] proposed a Markov model to describe the evolution process of particle fragmentation. However, it is still a challenge to construct a quantitative relationship between calcareous sand particle breakage and macroscopic mechanical behaviors.

As a numerical method suitable for the mechanical behaviors of granular materials, the discrete element method has been used to simulate the mechanical behaviors of calcareous sand. Initially, discrete elements method was used to simulate the friction behaviors between granular materials. The non-bonded contact model can reflect the behaviors of collision, friction and slippage between particles. However, for soils and rock materials, there is a certain bonding force between particles. If the non-bonded contact models are adopted, the mechanical characteristics cannot be reflected. As a method to model the particle-particle cementation, Potyondy and Cundall [33] proposed a parallel bond model (PBM) to account for the effect of bonding between particles. Shen et al. [34] built a particle breakage model of calcareous sand treated with MICP method based on discrete element method. The laws of crack propagation, crack distribution and the effect of particle breakage were studied. Kuang et al. [35] studied the impact of particles size on the crushing behaviors of single calcareous sand through single particle crushing tests. It found that the particle crushing strength data well met the Weibull statistical model. Lu et al. [36] established a three-dimensional discrete element model of calcareous sand particle breakage. The development of relative breakage rate and the evolution law of coordination number, porosity and sliding contact ratio were analyzed. The results showed that the three-dimensional model considering particle breakage could better reflect the micromechanical properties of calcareous sand. Yu et al [37] used the discrete element method to build a calcareous sand particle breakage model. The evolution characteristics of calcareous sand breakage laws were revealed during the consolidation and shear process. The influence of particle breaking characteristics on shear strength was discussed in details. To reflect the real process of particle breakage during the loading process, there are two numerical simulation methods. The first method is to represent single calcareous sand particle by cementing particle clusters. When the cementation strength between particles in the clusters reaches, the cementation bond would break to achieve the purpose of simulation [38–41]. This method can simulate the particle breakage very well, but the calculation amount is so large. The second method is called Fragment replacement method (FRM), which sets the particle breakage criterion in advance. Once the load on the particle exceeds its bearing limit, the particle is replaced by multiple smaller fragments [42–46]. The particle breakage simulation method based on FRM can reflect the mechanical response of particle assemblage to a certain extent. But the construction of fragment replacement model is subjective and lacks the support of physical test data and theoretical method. The existing discrete element simulation studies on particle breakage only focus on the decomposition of particles into several parts, ignoring that the contact mechanical properties between particles. The contact mechanical properties between particles have changed a lot during particle breakage. In the process of discrete element numerical simulation, the contact relationship between particles has an important impact on the simulation results. However, the existing contact models can not take particle breakage characteristics into account.

Therefore, based on the existing linear bond contact model and the binary media theory, this paper proposes a nonlinear binary medium bond contact model which can consider particle fragmentation. The established contact model uses C++ programming to generate a dynamic link library file (.dll) that can be called by PFC2D software. Then, a series of discrete element numerical simulations of calcareous sand were carried out using the established binary-medium bond contact model. The effects of damage parameters on stress-strain curves and strength were discussed based on the simulation results. The evolution laws of effective coordination number, fabric anisotropy and force chain number were analyzed. The relationships between macroscopic mechanical behaviors and meso-structure of calcareous sand were built.

## 2. Binary medium bond contact model theory

### 2.1. Linear bond contact model

**Fig 1:**
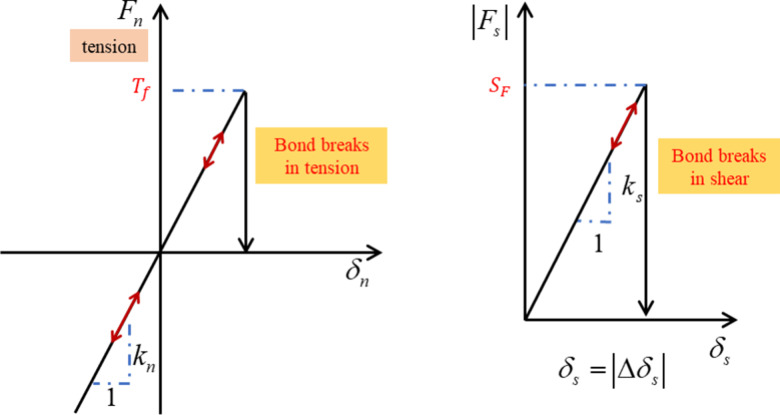
Schematic diagram of linear bond contact model.

The linear bond contact model (Fig 1), proposed by Potyondy [47], represents the bonding behaviors between particles based on the linear contact model. It is considered that the contact between particles can resist a certain tensile action in the normal direction of contact and a certain shear action in the tangential direction of contact. If the external force reaches the tensile strength of the contact, the bond between the particles will fail, resulting in no longer any contact force between the particles. If the external force reaches the shear strength, the bond between the particles will fail. And, the linear bond contact model degenerates into the linear contact model. The bond elements resist the external action until the failure. When the bond element is completely destroyed, only the friction element remains. Then the friction elements bear the external load.

The contact force between particles can be decomposed into normal contact force and tangential contact force, expressed as,


$$F=F_{n}\hat{n}\;+\;F_{s}\hat{s}$$ (1)

where $\hat{n}$ and $\hat{s}$ are the unit direction vectors of the normal force and the tangential force, respectively.

The normal force-displacement relationship of linear bond contact model is calculate by Eq (2). When the normal force of the contact is below the tensile strength, the normal force-displacement conforms to the law of linear elastic. When the normal force of contact is greater than the tensile strength, the contact between particles fails. The both the normal force and tangential force of contact are equal to 0.


$$\{\begin{array}{l} F_{n}\left(t_{0}+\Delta t\right)=F_{n}\left(t_{0}\right)+k_{n}\Delta \delta_{n}F_{n}\leq T_{F}\\ F_{n}\left(t_{0}+\Delta t\right)=0,F_{s}\left(t_{0}+\Delta t\right)=0F_{n}>T_{F} \end{array}$$ (2)

where $t_{0}$ represents the current moment, $t_{0}+\Delta t$ represents the next moment, $\Delta \delta_{n}$ represents the normal displacement increment, $k_{n}$ represents the normal stiffness, $T_{F}$ represents the contact tensile strength, $F_{n}$ represents the normal contact force, and $F_{s}$ represents the tangential contact force.

The tangential contact force of the linear bond contact model is calculated as follows,


$$\{\begin{array}{l} F_{s}\left(t_{0}+\Delta t\right)=F_{s}\left(t_{0}\right)+k_{s}\Delta \delta_{s}F_{s}\leq S_{F}\\ F_{s}\left(t_{0}+\Delta t\right)=\mu F_{n}\left(t_{0}\right)F_{s}>S_{F} \end{array}$$ (3)

where $\Delta \delta_{s}$ represents the increase in tangential displacement, $k_{s}$ represents the tangential stiffness, and $s_{F}$ represents the shear strength.

When the tangential contact force is lower than the shear strength, the relationship between the tangential force and displacement follows the law of elasticity. when the tangential force exceeds the shear strength, the bond between the particles is broken. The linear bond contact model degenerates into the linear contact model.

### 2.2. Theory of binary medium bond contact model

**Fig 2:**
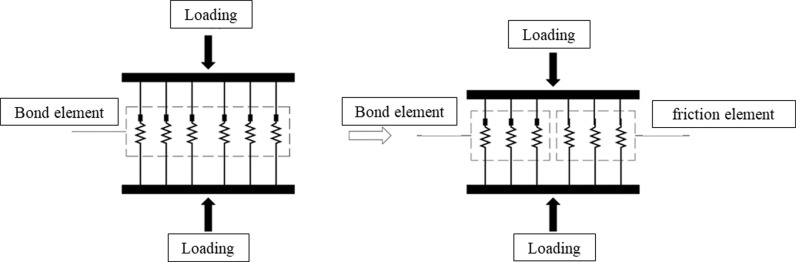
Binary media bonded contact model.

The Calcareous sand is a kind of geotechnical material with a certain cementation effect between particles. To fully consider the damage characteristics of particle cementation during loading, the particle contact between calcareous sand is abstracted into a bonding element with complete cementation effect and a friction element with cementation breaking (Fig 2). The bond element and friction element are independent of each other and have their own deformation characteristics. During the loading process, the bond element gradually breaks down and transforms into the friction element. The both elements resist the external action until the failure.

The force-displacement relationship of bond element contact is considered to be linear elastic, which can be expressed as:


$$F_{n}^{b}\left(t_{0}+\Delta t\right)=F_{n}^{b}\left(t_{0}\right)+k_{n}^{b}\Delta \delta_{n}$$ (4)

where $F_{n}^{b}$ represents the force on the cemented element contact, $\Delta \delta_{n}$ represents the increment of normal displacement, $k_{n}^{b}$ represents the normal stiffness of the bond element contact.

The relationship between tangential force and displacement for the bond element contact is:


$$F_{s}^{b}\left(t_{0}+\Delta t\right)=F_{s}^{b}\left(t_{0}\right)+k_{s}^{b}\Delta \delta_{s}$$ (5)

where $F_{\text{s}}^{b}$ represents the tangential force applied to the cemented element contact, $k_{s}^{b}$ represents the tangential stiffness, and $\Delta \delta_{s}$ represents the incremental tangential displacement.

The normal force-displacement relationship for friction element contact is:


$$F_{n}^{f}\left(t_{0}+\Delta t\right)=F_{n}^{f}\left(t_{0}\right)+k_{n}^{f}\Delta \delta_{n}$$ (6)

where $F_{n}^{f}$ represents the normal force of the friction element, $k_{n}^{f}$ represents the normal stiffness of the friction element, $\Delta \delta_{n}$ represents the normal displacement increment.

The tangential force-displacement relation of friction element conforms to the Mohr Coulomb criterion. It is expressed as:


$$\{\begin{array}{l} F_{s}^{f}\left(t_{0}+\Delta t\right)=F_{s}^{f}\left(t_{0}\right)+k_{f}^{s}\Delta \delta_{s}\text{, }F_{s}^{f}\leq \mu F_{N}\\ F_{s}^{f}\left(t_{0}+\Delta t\right)=\mu F_{N}\text{, }F_{s}^{f}>\mu F_{N} \end{array}$$ (7)

where $F_{s}^{f}$ represents the tangential force of the friction element, $k_{s}^{f}$ represents the tangential stiffness of the friction element, $\Delta \delta_{s}$ represents the tangential displacement increment, *μ* represents the friction coefficient of friction element.

A variable of damage parameter is introduced into binary medium bond contact model, which represents the change process of the bond element into friction element. When the external load starts to apply, the external load is all borne by the bond element, and the breakage rate is zero at this time. As the external load increases, the bond element gradually destroyed, turns into friction element. The bond elements and friction elements bear the load together. When the bond elements are completely broken, and the breakage rate is 1 currently. The external load is all borne by the friction element. This paper considers that breakage rate is a function of normal displacement, expressed as:


$$\gamma =1-\exp\left(-\alpha {\left(\delta_{n}\right)}^{\beta }\right)$$ (8)

where *γ* represents the rate at which the bond element breaks, while *α* and *β* represent the model parameters.

In summary, the total force in the normal direction of particle contact can be expressed as:


$$F_{n}\left(t_{0}+\Delta t\right)=F_{n}^{b}\left(t_{0}\right)+\left(\left(1-\gamma \right)k_{n}^{b}+\gamma k_{n}^{s}\right)\Delta \delta_{n}$$ (9)

The total force in the tangential direction of particle contact can be represented as:


$$\{\begin{array}{c} F_{s}^{f}\left(t_{0}+\Delta t\right)=F_{s}^{f}\left(t_{0}\right)+\left(\left(1-\gamma \right)k_{s}^{b}+\gamma k_{s}^{f}\right)\Delta \delta_{s}\text{, }F_{s}^{f}\leq \mu F_{N}\\ F_{s}^{f}\left(t_{0}+\Delta t\right)=F_{s}^{f}\left(t_{0}\right)+\left(1-\gamma \right)k_{s}^{b}\Delta \delta_{s}\text{, }F_{s}^{f}>\mu F_{N} \end{array}$$ (10)

It is considered that with the increase of breakage rate, the number of bond elements decreases, which will lead to the attenuation of the normal tensile strength and shear strength The attenuation expression is as follows:


$$S_{F}=S_{F0}\cdot \exp\left(-\xi \cdot \gamma \right)$$ (11)

$$T_{F}=T_{F0}\cdot \exp\left(-\xi \cdot \gamma \right)$$ (12)

where $S_{F0}$ represents the initial contact shear strength, $S_{F}$ represents the current contact shear strength, $T_{F0}$ represents the initial tensile strength, $T_{F}$ represents the current tensile strength, and *ξ* represents the model parameter.

The proposed binary medium bond contact model contains the following model parameters. $k_{n}^{b}$ is the normal stiffness of the bond element, $k_{s}^{b}$ is the tangential stiffness of the bond element, $k_{n}^{f}$ is the normal stiffness of the friction element, $k_{s}^{f}$ is the tangential stiffness of the friction element, *μ* is the friction coefficient. $S_{F0}$ is the initial shear strength, $T_{F0}$ is the tensile strength, *α*, *β* are the model parameters contact with breakage rate, *ξ* is attenuation parameter of tensile strength and shear strength.

### 2.3. Development and validation of a binary medium bond contact model

**Fig 3:**
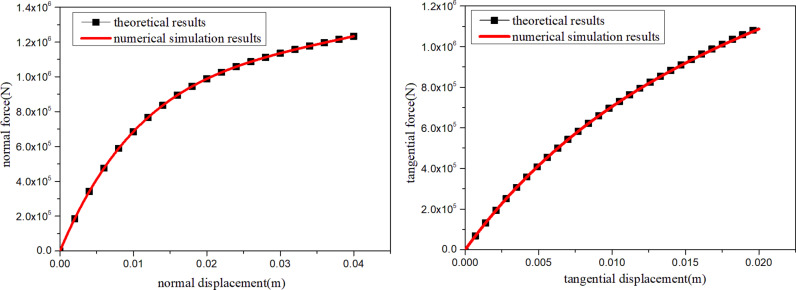
Comparison of numerical simulation and theoretical results.

To carry out discrete element numerical simulation, the proposed constitutive model was written into the particle flow program(PFC2D). To verify the proposed contact model is written correctly, the compression and direct shear simulation of a single contact were needed. Firstly, two balls with no overlap are generated. The single contact is generated between the two balls. The contact parameters for the single contact are assigned as shown in Table 1. During discrete element numerical simulation, the upper ball is fixed, and the lower ball is loaded with quasi-static velocity in the vertical downward direction or along the X-axis direction respectively. The normal force- displacement, tangential force-displacement of were recorded. The numerical results were compared with the theoretical calculation results, as shown in Fig 3. The verification results showed that the normal force-displacement and tangential force-displacement curves of the numerical simulation were in good agreement with the theoretical results. It confirmed the calculation accuracy of the proposed contact model. It indicated that the proposed binary medium bond contact model has been successfully written into the particle flow program (PFC2D).

**Table 1:** Contact parameters of single contact model.

$k_{n}^{b}$ /N	$k_{s}^{b}$ /N	$k_{n}^{f}$ /N	$k_{s}^{f}$ /N	$S_{F0}$ /N	$T_{F0}$ /N	*α* /	*β* /	*ξ*	*μ*
1.0e8	1.0e8	2.0e7	2.0e7	1.0e5	1.0e5	1.0	100	1.0	0.2

## 3. Discrete element numerical simulation of biaxial shear tests

### 3.1. Parameters calibration

**Fig 4:**
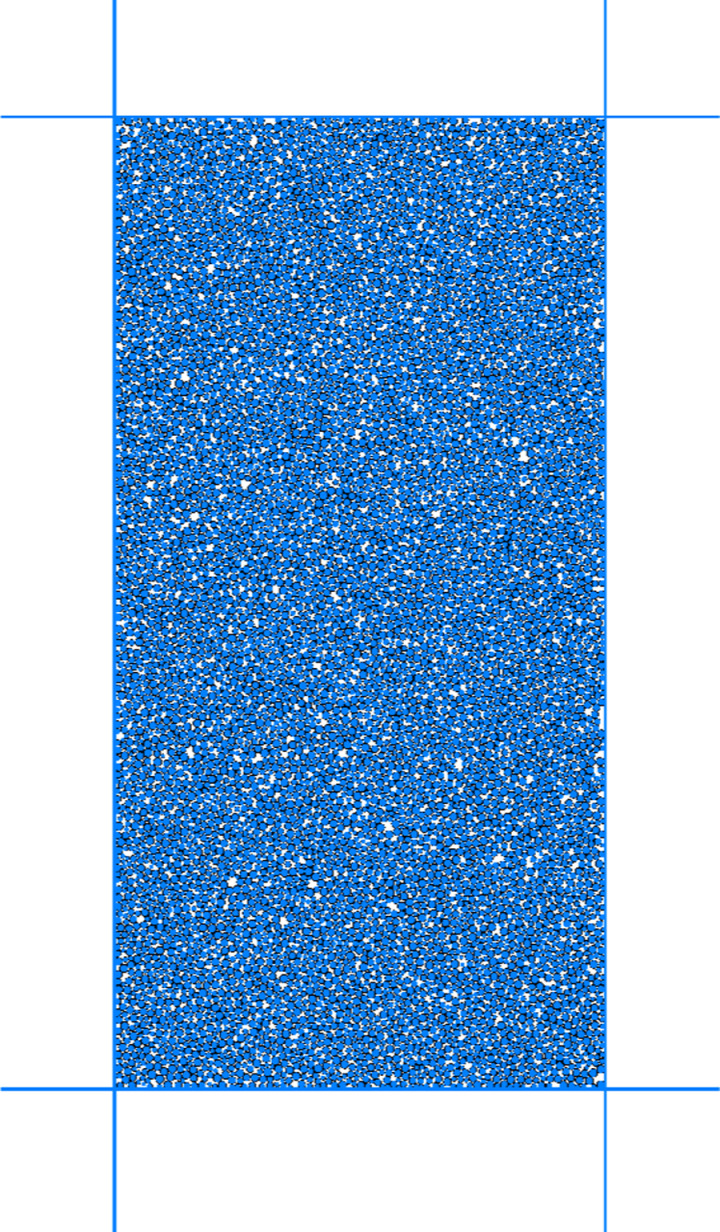
Calcareous sand biaxial shear discrete element calculation model.

**Fig 5:**
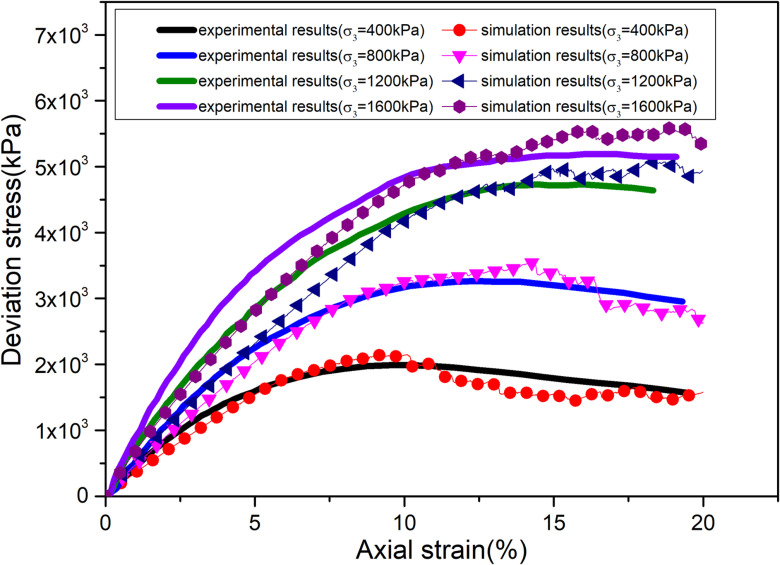
Comparison of test and simulation results [48].

**Fig 6:**
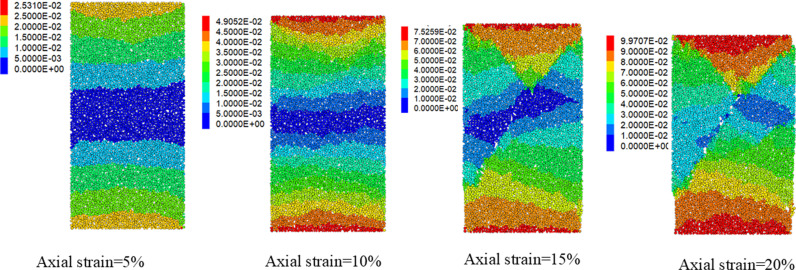
Displacement cloud image ($\sigma_{3}=0.4MPa$).

Before using the proposed binary media bond contact model to analyze the macro-micro mechanical properties of calcareous sand, it is necessary to calibrate the parameters of the proposed contact model. The parameters of the proposed contact model were calibrate by using the triaxial compression test results of calcareous sand under different confining pressures. The parameters of the binary media bond contact model contain the normal stiffness of the bond element $k_{n}^{b}$, the tangential stiffness of the bond element $k_{s}^{b}$, the normal stiffness of friction element $k_{n}^{f}$, the tangential stiffness of friction element $k_{s}^{f}$, the friction coefficient of the friction element *μ*, the initial shear strength $S_{F0}$, the initial tensile strength $T_{F0}$, the breakage rate parameter *α*, *β*, the tensile strength and shear strength attenuation parameter *ξ*. The normal stiffness $k_{n}^{b}$ and tangential stiffness $k_{s}^{b}$ of the bond element can be determined by the elastic phase of triaxial compression. The normal stiffness $k_{n}^{f}$ and the tangential stiffness $k_{s}^{f}$, and friction coefficient *μ*, can be determined by the late plastic flow phase. Additionally, the initial shear strength $S_{F0}$, initial tensile strength $T_{F0}$, breakage rate parameters *α*, *β*, strength attenuation parameter *ξ* can be determined by “trial-and-error” approach, which is adjusted parameters repeatedly until the simulation curve is close to the test data. Fig 4 shows the discrete element biaxial shear model with dimensions of 125 mm (height) *  61.8 mm (width). The model contains 5585 discrete element. The radius of the particles was between 4 mm–8 mm. The porosity of this model was set at 0.12. Fig 5 shows the comparison results of the stress-strain curves of the discrete element numerical results and the triaxial compression laboratory results [48]. The numerical results are in good agreement with the experimental results. It can also reflect the stress-strain curves change from strain-softening to strain-hardening with the increase of confining pressure. Fig 6 shows the displacement cloud image of particles with the increase of axial strain(0.4MPa). The obvious shear zone appears in the samples at the late loading stage. When the shear zone appears, the axial stress of the specimen gradually decreased. It proves that it is feasible to simulate the mechanical behavior of calcareous sand by using the proposed binary medium bond contact model. The calibration of mesoscopic parameters is shown in Table 2.

**Table 2:** The calibration of meso parameters of the discrete element model.

Confining pressure/kPa	$k_{n}^{b}$ /N	$k_{s}^{b}$ /N	$k_{n}^{f}$ /N	$k_{s}^{f}$ /N	$S_{F0}$ /N	$T_{F0}$ /N	*α* /	*β* /	*ξ*	*μ*
400	3.0e7	6.0e7	1.0e7	1.0e7	2.0e4	2.0e4	200	1.0	1.0	0.3
800	3.7e7	7.6e7	1.0e7	1.0e7	3.0e4	3.0e4	200	1.0	1.0	0.3
1200	4.0e7	8.0e7	1.0e7	1.0e7	4.5e4	5.5e4	200	1.0	1.0	0.3
1600	5.0e7	1.0e8	1.0e7	1.0e7	4.8e4	6.0e4	200	1.0	1.0	0.3

### 3.3. Analysis of micromechanical properties

#### 3.3.1. The effective coordination number.

The coordination number is directly related to the stability of the internal meso-structure of particle materials. The effective coordination number is used to describe the average contact number between the particles. The effective coordination number is different from the average coordination number. The no contact and only one contact between the particles are excluded. It can be expressed as:


$$C_{n}=\frac{2N_{c}-N_{b1}}{N_{b}-N_{b1}-N_{b0}}$$ (13)

where the $C_{n}$ represents the effective coordination number, $N_{c}$ and $N_{b}$ represent the total number of contacts and the number of particles, respectively. $N_{b0}$ represents the number of particles without contact, $N_{b1}$ represents the number of particles with only one contact.

**Fig 7:**
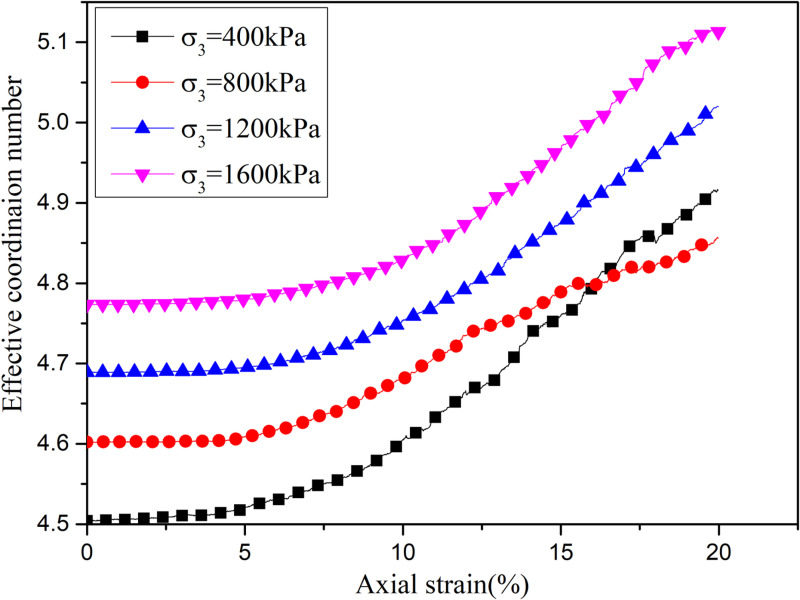
Effective coordination number under different confining pressures.

Fig 7 shows the evolution law of effective coordination number under different confining pressures. It shows that the initial effective coordination number increases with the increase of confining pressures. With the increase of axial strain, the average effective coordination number increases. This is because the friction elements continue to increase and bond elements continue to decrease with the increase of axial strain. As a result, the equivalent stiffness of the contact decreases, The particles become more compact, which leads to the coordination number continues to increase.

#### 3.3.2. Fracture number.

**Fig 8:**
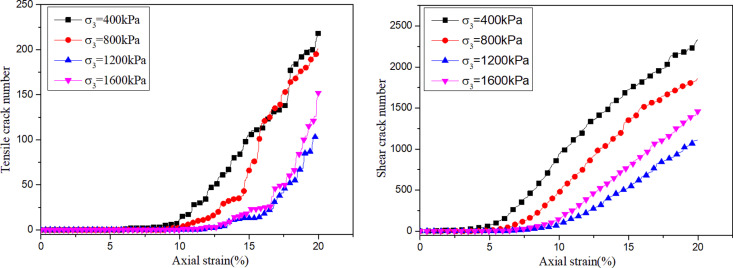
Number of fissures for different breakage parameters.

The cementation failure of binary medium bond contact model is monitored during the loading process. The cementation failure means that there are micro cracks between the particles. According to the failure mode, it is divided into shear crack and tension crack, so as to observe the mesoscopic failure process of Calcareous sand. Fig 8 shows the evolution laws of the number of internal micro-cracks in the biaxial shear under different confining pressures. It shows that tensile cracks and shear cracks in the sample continue to increase with the increase of axial strain. The shear cracks dominate, which indicates that the cementation failure of Calcareous sand particles is mainly caused by contact bonds reaching shear strength. With the increase of confining pressures, the number of tensile cracks and shear cracks decreases gradually under the same axial strain condition. It indicates that the confining pressure plays a certain role in inhibiting the development of cracks. The reduction of the cracks number makes the biaxial shear stress-strain curves transition from strain softening to strain hardening.

### 3.4 Fabric anisotropy

**Fig 9:**
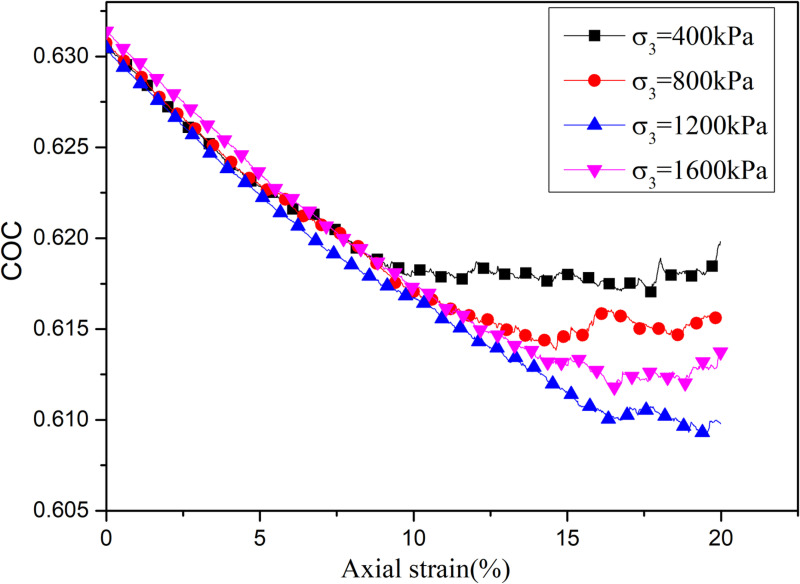
COC evolution under different confining pressure conditions.

Fabric Anisotropy is one of the main characteristics of granular materials, which is different from other continuous materials. To study the evolution law of contact normal direction, the contact orientation coefficient (COC) was defined as:


$$COC=\frac{1}{N}\sum_{i=1}^{N}\left|sin\theta_{i}\right|$$ (14)

where *N* represents the quantity of particle contacts, $\theta_{i}$ represents the angle between the particle contact normal and the horizontal direction. The COC reflects the main direction of the particle contact normal direction. The COC is close to 1, indicating that the particle contact normal direction is mostly vertical, and COC is close to 0, indicating that the particle contact normal direction is mostly horizontal. Fig 9 shows the evolution process of the normal coefficient COC of samples under different confining pressure. It shows that the contact normal coefficient decreases first and then tends to be stable with the increase of axial strain. In the initial stage, the axial pressure gradually increases with the increase of axial strain. The sample can be considered to be in the elastic stage without cracks inside the sample. When the specimen is in the elastic stage, the contact direction component increases along the horizontal direction and the normal coefficient of particle contact decreases gradually. Subsequently, the axial strain increased further, and the increase of axial stress decreased due to the continuous breaking of intergranular cementation. The sample enters into a state similar to plastic flow, so the anisotropy coefficient remained stable.

### 3.5. Force chains

**Fig 10:**
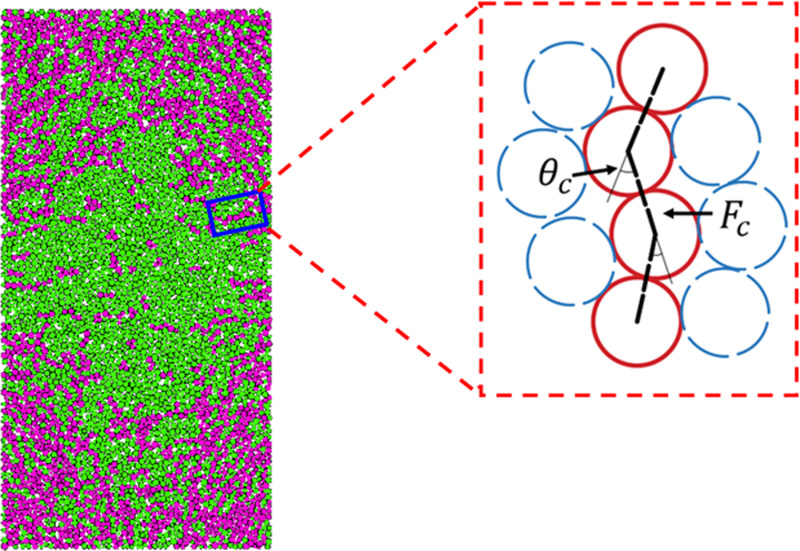
Identification of force chains.

In the particle system, the chain structure obtained by extracting the contact force between particles according to the chain formation criterion is called force chain. The force chain plays a key role in the force transfer between calcareous sand particles under external loads. Peters et al. [49] analyzed the particles by the principal stress method. To become a force chain, three conditions must be met at the same time (Fig 10): (1) The force chain consists of 3 or more particles, (2) The contact force of the force chain should be larger than the average contact force of the system, (3) The acute angle (*θ*) between the adjacent contact forces on the force chain should be smaller less than the specified threshold ($\theta_{c}$).

**Fig 11:**
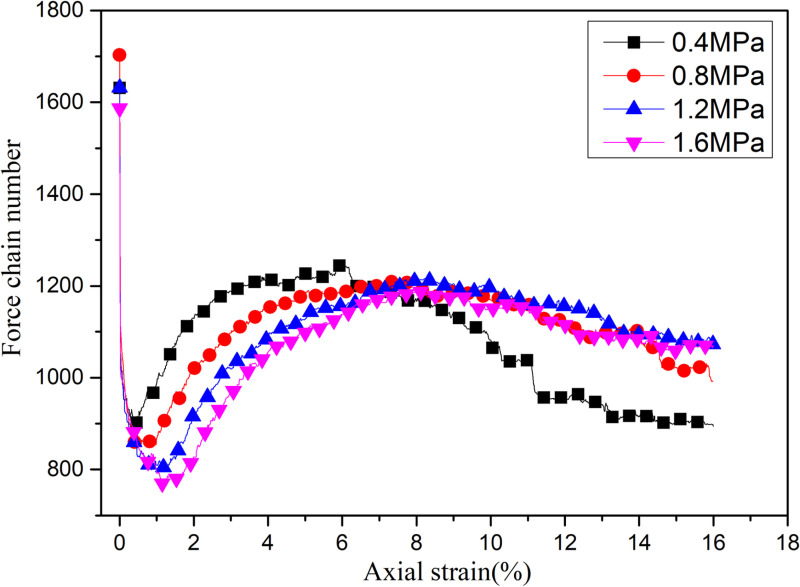
The law of chain number change.

Fig 11 shows the variation trend of the number of force chains with axial strain under different confining pressure. The number of force chains first decreases and then increases with the increase of axial strain. This is because in the isotropic state, the particles are evenly stressed. The more force chains are formed at this time. In the initial loading phase, a small number of high stress contacts gradually form resistance to external loads, resulting in a reduction in the number of force chains. With the increase of axial strain, the axial force gradually increases. The more force chains are needed to resist the external load. When the stress-strain curves enters the softening stage, the axial stress decreases and shear zone forms, causing the number of force chains to decrease again.

## 4. Conclusion

To reflect the cementation failure characteristics of calcareous sand particles, a discrete element contact model which can reflect the cementation characteristics of calcareous sand is derived by introducing the binary medium theory. A series of biaxial shear discrete element numerical simulations of calcareous sand were carried out by using this model to investigate the macro and micro mechanical properties of calcareous sand different confining pressures. The main conclusions of this paper are as follows:

(1) The calculation accuracy and applicability of the proposed contact model are verified by comparing the numerical calculation and theoretical analysis results of tensile and shear of single contact. The comparison results show that the proposed contact model is successfully written into the software. A series of numerical simulations of biaxial shear are carried out using the successfully written proposed contact model. The simulation results show that the proposed contact model can well reflect the shear mechanical properties of calcareous sand.
(2) The stress-strain curves gradually transitions from strain softening to strain hardening as the confining pressure increases. The cementation failure among the sample particles is mainly shear failure. Under the same axial strain condition, the tensile fracture and shear fracture in the sample gradually decrease with the increase of confining pressure.
(3) According to the mesoscopic parameters, the confining pressure plays a certain role in inhibiting the development of cracks. The reduction of the number of cracks makes the biaxial shear stress-strain curve transition from strain softening to strain hardening. When the stress-strain curves enters the softening stage, the axial stress decreases and shear zone form.

## Supporting information

S1 File Command stream-code. (ZIP)

S2 File Original data-data. (ZIP)
